# A Dockerized Approach to Dynamic Endpoint Management for RESTful Application Programming Interfaces in Internet of Things Ecosystems

**DOI:** 10.3390/s25102993

**Published:** 2025-05-09

**Authors:** Ebenhezer Mabotha, Nkateko E. Mabunda, Ahmed Ali

**Affiliations:** Department of Electrical and Electronic Technology, University of Johannesburg, Johannesburg 2092, South Africa; emabotha@gmail.com (E.M.); nkatekom@uj.ac.za (N.E.M.)

**Keywords:** RESTful, API, Docker, IoT

## Abstract

The growth of IoT devices has generated an increasing demand for effective, agile, and scalable deployment frameworks. Traditional IoT architectures are generally strained by interoperability, real-time responsiveness, and resource optimization due to inherent complexity in managing heterogeneous devices and large-scale deployments. While containerization and dynamic API frameworks are seen as solutions, current methodologies are founded primarily on static API architectures that cannot be adapted in real time with evolving data structures and communication needs. Dynamic routing has been explored, but current solutions lack database schema flexibility and endpoint management. This work presents a Dockerized framework that integrates Dynamic RESTful APIs with containerization to achieve maximum flexibility and performance in IoT configurations. With the use of FastAPI for asynchronous processing, the framework dynamically scales API schemas as per real-time conditions, achieving maximum device interaction efficiency. Docker provides guaranteed consistent, portable deployment across different environments. An emulated IoT environment was used to measure significant performance parameters, including functionality, throughput, response time, and scalability. The evaluation shows that the framework maintains high throughput, with an error rate of 3.11% under heavy loads and negligible latency across varying traffic conditions, ensuring fast response times without compromising system integrity. The framework demonstrates significant advantages in IoT scenarios requiring the addition of new parameters or I/O components where dynamic endpoint generation enables immediate monitoring without core application changes. Architectural decisions involving RESTful paradigms, microservices, and containerization are also discussed in this paper to ensure enhanced flexibility, modularity, and performance. The findings provide a valuable addition to dynamic IoT API framework design, illustrating how dynamic, Dockerized RESTful APIs can improve the efficiency and flexibility of IoT systems.

## 1. Introduction

The growth of the Internet of Things (IoT) has changed the way devices communicate and exchange data. With billions of interconnected devices forming the backbone of modern ecosystems, the demand for stable, flexible, and highly adaptable Application Programming Interfaces (APIs) has surged. These APIs must not only handle high volumes of data but also integrate with different systems, ensuring efficient and reliable performance in dynamic and growing IoT environments, as discussed by [1]. RESTful APIs, valued for their simplicity and compatibility with web technologies [2], are widely adopted in IoT systems. However, conventional RESTful APIs struggle with adaptability and scalability in dynamic IoT settings due to fixed endpoints and static configurations, leading to challenges in handling evolving device requirements [3,4].

To overcome these limitations, this paper proposes a Dynamic RESTful API framework suitable for IoT ecosystems, taking advantage of FastAPI’s asynchronous capabilities to dynamically generate endpoints and adapt database schemas based on device contexts, as demonstrated by its support for CRUD (Create, Read, Update, and Delete) operations. Compared to similar solutions, such as GraphQL, which offers flexible querying but requires complex schema definitions, or microservices-based APIs, which enhance scalability but introduce orchestration complexity [5,6], the proposed framework achieves high concurrency and low response times, as evidenced by its efficient handling of multiple simultaneous requests. These features are critical for applications like smart cities and healthcare monitoring, where real-time data processing is essential. Unlike API gateways, which may introduce latency and configuration overhead [7], the proposed framework minimizes setup complexity while supporting real-time adaptability.

Deployment of the suggested dynamic RESTful API is facilitated by Docker, which provides lightweight, portable environments for consistent performance across infrastructures. This addresses deployment challenges noted in other dynamic API systems [8], ensuring reproducibility and scalability. By integrating FastAPI’s performance advantages and Docker’s deployment efficiency, the proposed framework offers a strong solution for IoT applications requiring frequent updates and responsiveness, outperforming static REST implementations and gateway-based approaches in high-traffic scenarios [9].

## 2. Related Works

Dynamic APIs aim to address the limitations of traditional RESTful APIs, which struggle with fixed endpoints and static configurations in the face of the IoT’s dynamic requirements. However, implementing dynamic APIs introduces a host of challenges, including standardization, security vulnerabilities, deployment complexities, maintenance difficulties, and performance overheads.

### 2.1. Challenges in Dynamic API Implementations

Dynamic APIs are designed to adapt to changing requirements and workloads, but they face significant challenges that impact their effectiveness in IoT ecosystems. The lack of standardization across implementations is a primary concern, leading to inconsistency in API design, documentation, and usage. This variability complicates integration efforts, especially in heterogeneous IoT environments where devices employ different protocols, data formats, and communication systems [3]. For instance, without standardized endpoint structures, developers face challenges in ensuring interoperability between IoT devices and backend systems, resulting in increased development time and potential errors [4]. Security is another critical issue, as dynamic APIs, by their nature, expose configurable endpoints that can be vulnerable to attacks such as SQL injection, cross-site scripting (XSS), or unauthorized access if not properly secured [10]. These vulnerabilities are concerning in IoT systems, where compromised devices can disrupt critical applications like healthcare monitoring or industrial automation.

Deployment of dynamic APIs introduces additional complexities, as integrating these systems with existing infrastructure requires careful management of dependencies across different hardware, operating systems, and network configurations. In IoT contexts, where devices range from resource-constrained sensors to powerful edge servers, ensuring compatibility and performance consistency is important [6]. Maintenance further complicates matters, as frequent updates to accommodate new devices, protocols, or data requirements can lead to versioning conflicts, backward compatibility issues, and operational disruptions [11]. For example, updating an API to support a new IoT device type may break existing integrations, require manual software/application updates, and necessitate extensive testing [12]. The dynamic nature of these APIs introduces performance overhead, as real-time processing of configuration changes, such as generating new endpoints or modifying schemas, can degrade response times and throughput, particularly under high-traffic conditions [8]. Balancing flexibility with performance requires optimization strategies, a key focus of recent research [13,14].

### 2.2. Current Solutions and Comparisons

To address these challenges, several methods and architectures have been developed, each with distinct strengths and limitations. These include dynamic routing, API composition, middleware and interceptors, dynamic data filtering, API gateways, GraphQL, and gRPC. The proposed framework, which leverages FastAPI and Docker for dynamic endpoint generation and deployment, is compared against these solutions.

#### 2.2.1. API Gateways

API gateways serve as centralized entry points for managing API requests, offering features like authentication, rate limiting, load balancing, and security enforcement [15,16]. They are widely used in IoT systems to enhance scalability and standardize interactions between diverse devices and backend services. For example, gateways like Kong or AWS API Gateway can route requests to appropriate services, enforce access controls, and monitor traffic, making them suitable for large-scale IoT deployments. However, gateways introduce significant latency due to additional processing layers, which can be detrimental in real-time IoT applications, where low response times are critical. Configuring gateways to handle dynamic endpoints requires complex setup and maintenance, increasing operational overhead. In contrast, the proposed framework minimizes latency by directly managing endpoint generation within a lightweight FastAPI environment, as demonstrated by its efficient handling of CRUD operations across endpoints. The framework’s database-driven approach, storing endpoint configurations in separate tables, simplifies management compared to gateway-based systems, which often rely on static routing rules or complex policy definitions.

#### 2.2.2. GraphQL

GraphQL, an alternative to RESTful APIs, provides flexible querying capabilities, allowing clients to request specific data structures in a single request, reducing over-fetching and under-fetching issues common in REST [17,18,19]. This flexibility is advantageous in IoT systems with diverse data requirements, such as smart homes, where devices need tailored data payloads. However, GraphQL’s reliance on complex schema definitions, resolvers, and query validation introduces significant setup and maintenance overhead, making it less practical for resource-constrained IoT devices. In addition, GraphQL’s performance can degrade under complex queries, as resolving nested data structures requires additional server-side processing. The proposed framework, by contrast, leverages FastAPI’s asynchronous processing to dynamically generate RESTful endpoints without the need for schema definitions. The framework’s ability to handle CRUD operations efficiently ensures low response times and high concurrency, making it more suitable for IoT applications.

#### 2.2.3. gRPC

gRPC, built on HTTP/2 and protocol buffers, is designed for high-performance, low-latency communication, making it appealing for IoT applications requiring efficient data exchange, such as industrial automation [20]. Its compact binary format and support for bidirectional streaming enable faster data transfer compared to REST’s text-based payloads. However, gRPC’s reliance on HTTP/2 and specialized clients limits its compatibility with web-based IoT ecosystems, which predominantly use HTTP/1.1 and JSON. Additionally, gRPC’s complex setup, requiring protocol buffer definitions and client-side libraries, increases development overhead, particularly for resource-constrained devices [21]. The proposed framework, built on RESTful principles and FastAPI, ensures broad compatibility with web technologies while maintaining high performance, as evidenced by its asynchronous handling of multiple simultaneous requests. The framework’s lightweight design and Docker-based deployment make it more accessible and easier to integrate into diverse IoT environments compared to gRPC [22].

Niswar et al. [23] evaluated the performance of REST, gRPC, and GraphQL communication protocols within microservices using Redis and MySQL. The study found that gRPC offers the fastest response time, especially for nested data retrieval, due to its use of HTTP/2 multiplexing. However, REST demonstrates the lowest CPU utilization, making it a resource-efficient choice. These insights highlight REST as a balanced and reliable protocol for microservices environments, particularly when system efficiency and simplicity are priorities.

#### 2.2.4. Limitations of Existing REST Frameworks

While RESTful APIs remain a dominant approach in web and IoT systems due to their simplicity and compatibility, most popular frameworks, such as Flask, FastAPI, and Django, support dynamic routing at the URL level [24,25,26]. These frameworks allow developers to define endpoints with parameters (e.g., /device/<id>, /sensor/<type>), enabling flexible route matching. However, beneath this dynamic routing, the data models and logic are typically static, relying on hardcoded schemas and object-relational mappings (ORMs). This rigid structure poses challenges in IoT environments, where data schemas often vary across devices and evolve over time.

Manual intervention is usually required to update models, perform migrations, or adapt to new device data formats, limiting flexibility and introducing operational overhead. This issue becomes pronounced in heterogeneous IoT systems that demand schema adaptability and runtime flexibility. In addition, despite FastAPI’s support for asynchronous execution and automatic documentation generation, it still relies heavily on predefined Pydantic models, which are not inherently dynamic.

In contrast, the proposed framework introduces a schema-less, database-driven approach to endpoint generation. By storing endpoint configurations and data structure definitions directly in a dedicated metadata table, the framework allows for the dynamic registration and updating of API routes at runtime. This eliminates the need for code-level model changes and makes the system highly adaptable to new devices and data types. Coupled with Docker-based deployment and asynchronous handling, the framework not only addresses the limitations of conventional REST frameworks but also ensures low latency and high scalability in different IoT deployments.

### 2.3. Testing and Optimization Strategies

API testing encompasses a range of techniques, including unit testing, integration testing, and end-to-end testing, to validate functionality, performance, and reliability [27]. Unit tests verify individual API endpoints, while integration tests ensure smooth interactions between the API, database, and external systems. End-to-end tests simulate real-world IoT scenarios, such as thousands of devices sending simultaneous requests, to assess overall system behavior [28]. Performance testing, which measures metrics like throughput, response time, and resource consumption under varying loads, is critical for dynamic APIs in high-traffic IoT environments [13]. Tools like Jmeter (v5.6.2), Postman (v11.43.4), and LoadRunner (v2023) are widely adopted for performance testing. JMeter, an open-source tool, excels in simulating heavy loads to evaluate system strength across load testing, stress testing, and endurance testing, providing detailed insights into throughput, response times, and resource usage [29,30,31,32]. LoadRunner, with its advanced scripting and monitoring capabilities, is preferred for large-scale, enterprise-grade systems, offering granular performance analysis [33]. Recent advancements in black-box testing, which evaluates APIs without knowledge of their internal structure, have further enhanced reliability by systematically identifying vulnerabilities and performance bottlenecks, as demonstrated in RESTful API testing frameworks like RESTest. These methodologies are directly applicable to the proposed framework, which was evaluated using similar metrics.

Throughput, defined as the number of requests processed per unit of time, is a critical metric for IoT systems, where thousands of devices may generate continuous data streams [13]. Optimizing throughput requires efficient resource management, including CPU, memory, and network bandwidth, to handle concurrent requests without degradation. The proposed framework’s high throughput results from FastAPI’s asynchronous event loop, which processes requests concurrently without blocking, a strategy supported by recent research on API optimization [14]. Response time, which measures the latency between a request and its response, is equally important, as low response times are essential for real-time IoT applications like traffic management or patient monitoring [34]. Adaptive algorithms and load-balancing techniques, such as those implemented in FastAPI, significantly reduce response times by distributing workloads efficiently. Reliability, assessed through fault injection testing, ensures consistent performance under failure conditions, such as network delays or server crashes [19]. The framework’s high reliability is supported by Docker’s isolated environments, which minimize external interference and ensure consistent behavior [22].

Scalability, the ability to handle increased loads by adding resources, is a cornerstone of dynamic API design for IoT. Microservices architectures enhance scalability by allowing individual services to scale independently, a strategy widely adopted in IoT systems [5]. However, the proposed framework achieves similar flexibility without microservices’ complexity by leveraging Docker’s containerization and FastAPI’s lightweight architecture, as demonstrated by its performance under varying workloads (Section 4.4). Containerization technologies, particularly Docker, have become integral to API testing and deployment, providing isolated environments that ensure consistent results across development, testing, and production stages [35]. Docker’s ability to encapsulate the framework and its dependencies addresses deployment challenges noted in prior work. Recent advancements in AI and machine learning have further transformed API testing by enabling tools to detect bottlenecks, predict performance issues, and optimize parameters dynamically based on real-time feedback [36]. For example, AI-driven testing tools like those described in [36] can adjust JMeter test scripts to focus on high-risk endpoints, improving the accuracy of performance evaluations. These advancements are particularly relevant for IoT systems, where unpredictable workloads require adaptive testing strategies.

## 3. Materials and Methods

**Figure 1:**
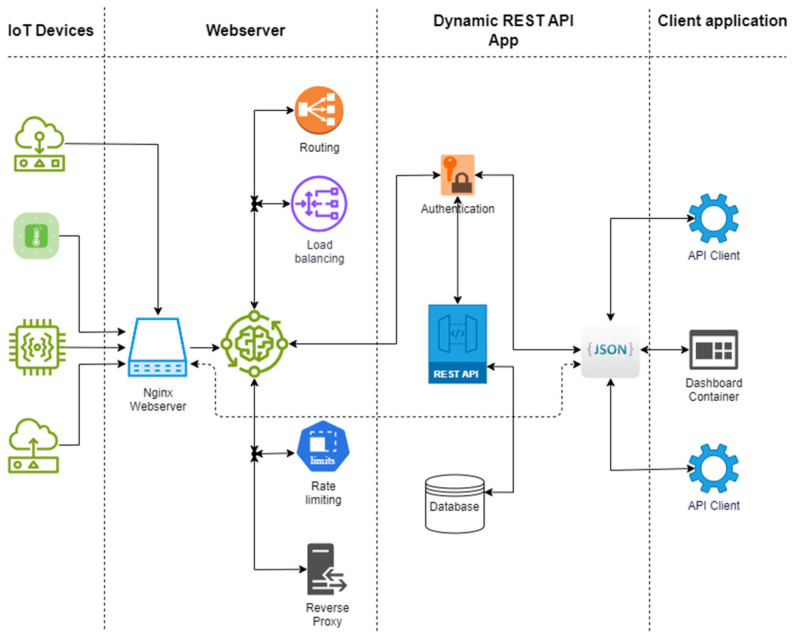
Architectural design.

This section outlines the method for developing and deploying the dynamic RESTful API framework, emphasizing framework architecture, RESTful API design, data storage, deployment, and usage. The development process adheres to best practices to ensure scalability, adaptability, and maintainability for successful IoT system deployment and operation. The framework architecture is illustrated in Figure 1.

### 3.1. Framework Design

The architecture of the proposed solution is based on microservices and containerization principles, allowing for modularity, scalability, and efficient resource management. The system consists of two primary Docker containers: a webserver container and a RESTful API container. The webserver is implemented using Nginx, functioning as a reverse proxy to handle incoming requests, enforce rate limits, and direct traffic to backend services. The RESTful API container hosts a FastAPI application integrated with PostgreSQL for data storage and dynamic schema management.

### 3.2. Docker Containers

To support modularity and portability, each component of the framework was containerized using Docker. This provides a lightweight and portable method for encapsulating program components, ensuring consistency and reproducibility across various environments, as detailed by [37]. The containerized architecture comprises two main services: a webserver container running Nginx and an API container running a FastAPI application with a PostgreSQL database. These containers work independently but communicate over mapped ports to ensure proper routing and data handling.

#### 3.2.1. Webserver Container

The webserver container acts as the entry point to the framework and is responsible for routing incoming HTTP requests. Built from the official Nginx image on Docker Hub, this container includes a custom configuration that enables rate limiting and request forwarding. It serves as a reverse proxy, directing requests from IoT devices and client applications to the appropriate backend services. This configuration enhances performance and security, with Nginx selected due to its proven speed and efficiency over alternatives such as Apache [38]. The container exposes port 80, allowing external clients to interact with the system using standard HTTP protocols.

#### 3.2.2. Dynamic RESTful API (Interface) Container

The API container is built on Ubuntu 22.04, selected for its stability and Docker compatibility, based on recommendations from [39]. This container includes both the FastAPI application and a PostgreSQL database, which work in tandem to support dynamic schema creation and data storage.

During the image build process, system packages and Python dependencies are installed in non-interactive mode to streamline installation. The application code is organized within the /app directory, following a clean project structure. Environment variables are configured for secure and efficient PostgreSQL database management within the container. Critical system packages such as iproute2 are also installed to enable proper network and container management.

The container exposes port 8000 for handling API traffic and port 5432 for PostgreSQL connections. These port mappings ensure proper interaction between services within and outside the containerized environment. SQL initialization scripts and FastAPI startup commands are used to configure the application during runtime, ensuring both the database and the API server are deployed successfully.

This containerized design follows principles outlined in [40], supporting isolated, efficient, and scalable deployment of RESTful APIs in dynamic IoT environments. The architecture facilitates reliable operation and maintainability, providing a robust foundation for ongoing development and integration with external systems.

### 3.3. RESTful API Application

Python was chosen for the dynamic RESTful API application for its reliability, efficiency, and broad library and framework ecosystem. Python’s versatility and ease of interface with multiple systems make it an excellent tool for API development. FastAPI, a modern and high-performance web framework for developing APIs with Python 3.6+, was chosen due to its excellent speed and broad feature set. FastAPI generates automatic interactive API documentation utilizing OpenAPI and JSON Schema, greatly improving the development and debugging processes. According to [25], this automatic documentation supports fluid client–server connection by offering a clear and interactive interface for API endpoints (interface), allowing developers to better understand and use API functions.

FastAPI’s compatibility with Python’s asyncio library enables it to handle several concurrent requests efficiently, optimizing performance under large loads, as advocated by [41]. This asynchronous feature is crucial for applications that require several concurrent interactions, such as IoT systems where devices frequently send and receive data.

### 3.4. Database and Storage

The database for the RESTful API application was carefully selected based on parameters such as scalability, performance, and ease of interaction with the chosen technology stack. According to the comprehensive study detailed in [42], PostgreSQL emerged as the recommended database management system due to its extensive feature set, durability, and strong support within the Python environment. PostgreSQL’s solid support for the JSONB data type played an important role in the decision to use it, as it enables flexible schema design that can accommodate various data patterns found in dynamic API applications. This versatility is essential for addressing the numerous and changing data formats encountered in IoT and other dynamic settings.

Data storage in PostgreSQL is customized to the application’s operational requirements, such as CRUD operations across many endpoints and authentication processes. The database schema, especially for user information and endpoint structure, has been carefully designed to ensure data integrity and efficient querying. This design adheres to relational database ideas while using PostgreSQL’s additional features to perform complex queries and transactions. PostgreSQL’s transactional capability enables ACID (Atomicity, Consistency, Isolation, Durability) compliance, which is vital for maintaining data consistency and dependability in critical transactional activities, as recommended by [43]. Furthermore, PostgreSQL’s FastAPI compliance is implemented through asynchronous database access, which enhances performance by efficiently managing concurrent queries. This method significantly increases the API application’s scalability, ensuring responsiveness and stability under changing workloads.

### 3.5. Deployment

**Figure 2:**
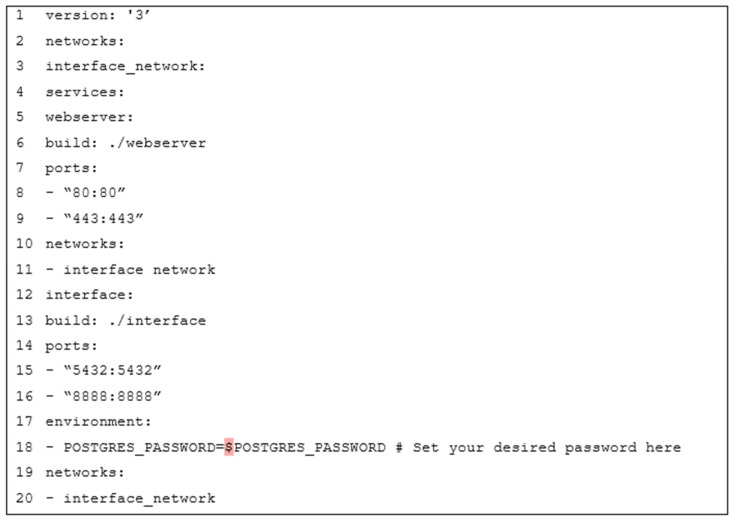
Docker-compose file.

The framework was deployed and tested on a Virtual Private Server (VPS), which provided consistent resource allocation and control. This ensured a reliable and isolated environment for container management and performance evaluation. The VPS specifications are listed in Table 1:

The deployment process uses Docker Compose to automate container initialization. Docker Compose, as demonstrated in [44,45,46], makes it easier to develop and deploy multi-container Docker applications. Figure 2 shows an overview of the docker-compose.yml file, defining the needed services and configurations.

The docker-compose.yml file in Figure 2 defines two primary services:

#### 3.5.1. Webserver Service (Webserver)

The Webserver Service is responsible for building and configuring the webserver container. It uses the Dockerfile located in the ./webserver directory to create the webserver container image. The service exposes two critical ports: port 80 on the host is mapped to port 80 on the container for HTTP traffic, and port 443 on the host is mapped to port 443 on the container for HTTPS traffic. This ensures that the webserver can handle both standard and secure web requests. Additionally, the service connects to the interface_network, enabling seamless communication between the webserver and other services within the same network, which is essential for a cohesive and integrated system.

#### 3.5.2. Dynamic RESTful API Service (Interface)

The Dynamic RESTful API Service handles the creation and configuration of the interface container. It builds the interface using the Dockerfile located in the ./interface directory, generating the interface container image. The service maps port 5432 on the host to port 5432 on the container, allowing access to the PostgreSQL database, and port 8888 on the host to port 8888 on the container for the FastAPI application. Environment variables, such as POSTGRES_PASSWORD, are set to configure the PostgreSQL database password, ensuring secure and customizable database access. Like the Webserver Service, this service also connects to the interface_network, facilitating communication between the interface and other services within the network, which is crucial for maintaining a unified and efficient system architecture.

### 3.6. Performance Evaluation

The dynamic RESTful API’s performance evaluation methodology was based on an organized process that guarantees thorough testing in all areas, from security to functionality. The main techniques and procedures for assessing the dependability and performance of the API are described below.

#### 3.6.1. Authentication and Authorization

The authentication and permission testing for the Dynamic RESTful API was developed to ensure that only valid and authorized users may access and use the API securely. The testing process started with user registration. When a user registers, they provide a valid email address and a password via the registration API endpoint. When the user successfully registers, the system sends a validation email with a unique link to their email address. The link in the email must be clicked to verify the email address and activate the account. The testing procedure ensured that the validation email was delivered correctly, and logs were reviewed to ensure that the email was sent successfully. Special scenarios, such as processing incorrect email addresses and managing requests for resending validation emails, were also tested to ensure the system’s reliability

Once the user’s email address has been verified, the user is authorized to log in. On login, the user submits their email address and password, and after successful authentication, the system generates a unique api_key for that user. This api_key acts as a token and must be supplied in the header of any subsequent API calls to authenticate the user and grant access to the API’s endpoints. The security of this process was validated by ensuring that the api_key was produced appropriately upon login, securely maintained, and properly invalidated upon logout or session expiration. Unauthorized access attempts, such as those with invalid or expired api_keys, were also assessed to ensure that the API delivered suitable error answers and refused access to secure resources.

#### 3.6.2. Status Code Validation

The status code verification process was carried out to determine the API endpoints’ responses and reliability in handling various types of queries. This step is important for ensuring that the API returns the correct HTTP status code based on the kind of request and its results. The purpose of testing several endpoints with a variety of valid and invalid inputs was to ensure that each endpoint behaved as expected under different scenarios, such as successful operations, unauthorized access, resource unavailability, and validation failures.

Each endpoint was subjected to several test cases that simulated real-world conditions. For example, POST requests were tested to ensure that data were created and sent, and GET requests were reviewed to retrieve data correctly. PUT and DELETE requests were used to evaluate the handling of updates and deletions. These tests validated the API’s ability to handle errors and provide correct responses.

The testing process validated that the API followed traditional standards by returning accurate status codes, which are important to client–server communication. Correct status codes inform clients about the success or failure of requests, allowing them to implement effective error handling and improve the user experience. This method is also important for identifying possible difficulties with data validation, resource management, and security, as well as providing insights into areas for future optimization.

#### 3.6.3. Functional Testing

To ensure that every API endpoint carries out its intended functions as intended, functional testing is the main emphasis of the assessment process’s initial step. In this process, Postman was used to create test cases for each endpoint, including POST, GET, PUT, and DELETE. Every endpoint undergoes extensive testing to guarantee that it can carry out necessary tasks, including setting up, obtaining, updating, and removing dynamic interfaces.

During functional testing, response validation was important, and therefore the Nginx rate limit was not considered in this test. The API responses were compared to the expected status codes and payloads, as provided in the API documentation. This phase verified that the API consistently returned the appropriate data structure and values. The API’s error-handling capabilities were tested using incorrect inputs and exceptions. The system’s ability to return relevant error messages and gracefully manage exceptions was tested to verify reliability in real-world circumstances.

#### 3.6.4. Load Testing

A series of load tests were conducted to evaluate the performance of an Nginx server hosting an API, configured with a rate-limiting rule defined as limit_req_zone $binary_remote_addr zone = mylimit:10 m rate = 10 r/s;, which restricted each IP address to a maximum of ten requests per second using a 10 MB memory zone for tracking client requests. To obtain accurate application performance results under high load, the rate limit was intentionally bypassed during the test. The primary objective was to assess the server’s underlying capacity to handle traffic beyond the rate-limiting threshold, focusing on the API’s performance metrics, such as response times, throughput, error rates, and latency under stress conditions. Bypassing the rate limit allowed the test to simulate a scenario where the server experienced excessive traffic, providing insights into the application’s scalability and stability without the constraints of the rate-limiting mechanism, which was later confirmed by the observed throughput exceeding the 10 requests per second limit.

JMeter was utilized to generate controlled traffic and monitor key performance metrics over a duration of 141 s. The test configuration included the following parameters: a single thread to simulate one user, a ramp-up period of 0 s, an infinite loop count to ensure continuous requests, and a target throughput of 600 requests per minute, equivalent to approximately 10 requests per second. Although the target throughput aligned with the rate limit, the bypass ensured that JMeter could exceed this threshold, as evidenced by the initial throughput of 67.1 requests per second, allowing the test to stress the application beyond the configured restriction.

The collected metrics were logged in a CSV file for detailed analysis, capturing parameters such as timestamps, response times, response codes, and throughput. This methodology enabled a comprehensive assessment of the API’s performance under a high load scenario, offering insights into its behavior when the rate-limiting mechanism was disabled. The test design also facilitated the identification of performance bottlenecks and error patterns, providing a foundation for evaluating the application’s resilience and scalability under conditions exceeding typical operational limits.

#### 3.6.5. Response Time Testing

Response time testing was conducted to evaluate the API’s performance under typical load scenarios, focusing on the latency experienced by users during standard operations. The tests were performed using JMeter, configured with 10 concurrent threads to simulate multiple users, a ramp-up period of 10 s, and a total of 1000 requests per endpoint, targeting the /login, /data, and /status endpoints. The load was distributed evenly across these endpoints to mimic real-world usage patterns, with each thread generating requests at a rate of 1 request per second, resulting in a total throughput of 10 requests per second. The tests were run for 100 s to ensure a sufficient sample size for analysis. Key metrics, including average response time, minimum and maximum response times, and error rates, were logged for each endpoint. These tests provided insights into the API’s responsiveness under typical load conditions, with detailed results presented in Section 4.4.2.

### 3.7. Data Collection

The data were collected using a Raspberry Pi 4 and an ESP8266 NodeMCU microcontroller, both equipped with a DHT11 temperature and humidity sensor. The Raspberry Pi was also used to monitor system metrics, including CPU, memory, and disk consumption. Both devices have Wi-Fi connectivity, which allows them to send HTTP requests and communicate with a server or API for data transport and storage. This setup supported the study’s entire data collection, ensuring that a variety of external and system performance variables were collected and analyzed.

### 3.8. Trade-Offs of Dockerized APIs

The dynamic RESTful API framework presented in this study uses Docker containerization for flexibility and scalability in IoT applications, deployed on a Virtual Private Server with 2 virtual CPUs (vCPUs), 2 GB RAM, a 40 GB SSD, and a Ubuntu 22.04 LTS. Running Dockerized APIs on a VPS introduces specific trade-offs compared to bare-metal setups and virtual machines (VMs) in terms of resource utilization and performance.

Docker’s lightweight containerization reduces overhead by sharing the host OS kernel. Load testing (Section 4.4.4) of the Dockerized setup (NGINX, FastAPI, PostgreSQL) achieved an average throughput of 9.83 requests/sec across 4732 requests over 141 s, peaking at 67.1 requests/sec in the first minute. However, the VPS’s 2 vCPUs and 2 GB RAM led to contention during this peak, causing response time spikes of up to 760 ms due to memory swapping and CPU limits. The SSD mitigated I/O bottlenecks, but the VPS’s virtualization layer added slight overhead, as the hypervisor managing the VPS (e.g., KVM or VMware) consumes some CPU and memory resources, reducing availability for Docker containers compared to a bare-metal setup. A bare-metal deployment would eliminate both the VPS hypervisor and Docker orchestration overhead, potentially stabilizing response times and increasing throughput, but it lacks Docker’s scalability and the VPS’s isolation benefits for the IoT, enhanced in this setup with NGINX and Docker Compose.

Compared to a nested VM approach, running additional VMs inside the VPS, Docker is more efficient. A nested VM would exacerbate resource constraints, as the VPS’s 2 GB RAM and 2 vCPUs would be further divided, likely causing excessive swapping and reducing throughput well below 9.83 requests/s. Nested VMs also introduce higher latency due to multiple virtualization layers, slowing scaling for IoT workloads. Running Docker on a VPS strikes a balance, leveraging the VPS’s isolation and cost-effectiveness while maintaining Docker’s scalability, though the limited resources highlight the need for careful optimization. Future work will focus on optimizing container configurations, exploring VPS upgrades with more vCPUs and RAM, and testing on bare-metal to compare performance in diverse IoT scenarios.

## 4. Results and Discussion

This section presents the outcomes of the developed system, focusing on the RESTful API application, database functionalities, and data collection capabilities using IoT devices.

### 4.1. Dynamic RESTful API

The RESTful API application created with FastAPI has proven to be effective and functional in dynamic settings. FastAPI is highly efficient and offers JSON Schema and OpenAPI-based automatic API documentation. By utilizing its asynchronous capabilities, the API effectively manages multiple requests at once, maximizing efficiency even in situations of high traffic. The RESTful principles are closely followed by the API, allowing CRUD activities across numerous endpoints, including authentication features for Sign Up and Login. All endpoints ensure smooth client–server communications by responding effectively and adhering to HTTP status code standards. Table 2 contains a list of all the dynamic API’s endpoints, and the sections that follow provide the details of each endpoint’s applicable functionality and methods.

The tables below detail the specific requests and responses associated with each endpoint, providing insights into the API’s interaction protocols:

#### 4.1.1. Adding an Interface

Table 3 illustrates the request and response information on how to add a new interface.

#### 4.1.2. Retrieve Interface

The user’s dynamic interfaces can be retrieved using the GET method. The application pulls all the interfaces from the database and verifies the user upon getting a request. An error message is sent back if failures occur during this process. Table 4 illustrates the request and response information for this method.

#### 4.1.3. Update Interface

The “/dynamic” route’s PUT method functions similarly to the POST method, except that, instead of generating a new endpoint, it updates the table with new fields or deletes ones that are not in the updated payload. The revised payload will be added to the stored structure that is used for validation. The update request and response are illustrated in Table 5.

#### 4.1.4. Delete Interface

The “/dynamic” route’s DELETE function will remove the particular interface. The endpoint uses an API key to authenticate the user upon receiving a request. A suitable error message is returned in the event that authentication fails. To confirm that the interface is present, the endpoint then extracts the interface identity from the request payload. Next, the database is accessed to retrieve the interface data that matches. Subsequently, the interface entry is deleted and the associated table is extracted from the database. Table 6 illustrates the details of the delete method.

#### 4.1.5. Add Interface Data/Records

This method is used to add the device records or history. The URL string that was provided when the endpoint was created can be used to publish additional entries or data. This can be accomplished by sending a POST request to the “/dynamic/<URL>” route. Prior to returning the data, the application verifies the user’s details. Table 7 illustrates the request and response information.

#### 4.1.6. Get Interface Data/History

The interface records/history can be retrieved by sending the GET request to the “/dynamic/interface/<URL>” route. Prior to returning the data, the application verifies the user’s details. Table 8 illustrates the request and response information.

### 4.2. Container Implementation

The Webserver and the Interface are the two main containers used in the architecture. As explained in the methodology section, the Webserver handles the requests, while the Interface is the primary container enabling API interactions. The Interface container offers flexibility and efficiency, which can be used independently of any common API client. The outcomes of the containers produced by using the method are listed below:

#### 4.2.1. Configuration of the Webserver Container

The webserver container was built using the official Nginx image from Docker Hub, but its default configuration was modified to include custom rate limitations and routing tailored for the application. The configuration sets a request rate limit of 10 requests per second using the $binary_remote_addr variable. Client request headers, such as Host, X-Real-IP, and User-Agent, are preserved and forwarded to the backend service. The root directory serves static files from /usr/share/nginx/html, while API requests to /interface/ are proxied to an external service running on port 8888, with rate limiting applied. Custom error handling is also implemented, including a dedicated 404 error page that attempts to resolve missing resources and a generic error page for server-side failures. This setup enhances security, load management, and user experience within the application.

#### 4.2.2. Interface Container

The Interface container’s capability to support Internet of Things (IoT) operations was successfully demonstrated through its deployment and evaluation using a dynamic, RESTful API. The assessment focused on three key areas: security, reliability, and flexibility. Key findings emerged when the framework was applied to create dynamic interfaces for ESP8266 and Raspberry Pi devices. In terms of security and user authentication, the system effectively authenticated users via Sign-up and Login endpoints, while the Nginx webserver enforced rate-limiting policies to prevent misuse and ensure fair resource usage. Regarding the creation of dynamic interfaces and endpoints, the process involved generating new interfaces using specified fields such as “field_name” “field_type” “trendable” and “mandatory” for data validation. The system stored endpoint structures, created corresponding database tables, and verified user credentials to support future modifications. Additionally, the implementation of clear error notifications significantly improved the reliability and user experience of the dynamic endpoint creation feature. These findings underscore the framework’s ability to deliver secure, adaptable, and user-friendly solutions for IoT applications.

### 4.3. Framework Use Case: ESP8266 and Raspberry Pi Interfaces

To demonstrate the practical application of the Dynamic RESTful API framework, two new dynamic interfaces were added for ESP8266 and Raspberry Pi devices. The Raspberry Pi interface, accessible via /raspberrypi, monitors system metrics like CPU, RAM, and disk usage, with each field (e.g., “cpu”, “disk”, “ram”) set as an integer, marked as trendable and required. Similarly, the ESP8266 interface, available at /esp, collects temperature and humidity data from a weather station sensor, with both fields defined as integers and also marked as trendable and required. These interfaces store raw information in separate database tables, which are used for request and response validation. The stored data ensures that incoming requests adhere to the correct data types and parameters, enabling the system to return appropriate error messages when invalid data are submitted, thus maintaining data integrity and system consistency.

The raw interface information is stored in a separate table. This information is used to verify the request responses and return appropriate errors when necessary. For example, if a user tries to update the interface or add a new record, the application will use this information to validate each parameter and its type. Table 9 displays the raw dynamic table information from the database.

#### 4.3.1. Hardware Setup

To test the framework, temperature and humidity measurements from the DHT11 sensor were supplied to the Esp8266. The sensor was connected to ground and 3.3 V VCC, and pin D5 of the ESP8266 microcontroller was particularly used to record temperature readings. The collected data were then sent over the HTTP protocol to the specified endpoint, “/dynamic/esp”.

**Figure 3:**
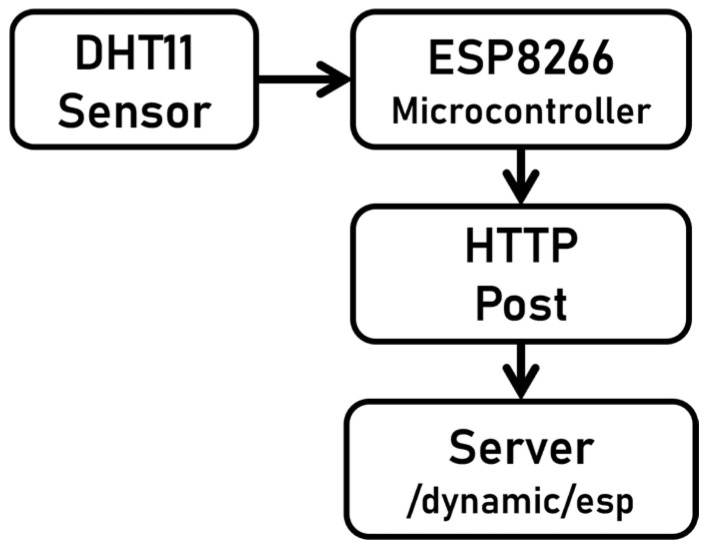
ESP8266 NodeMCU hardware connection.

**Figure 4:**
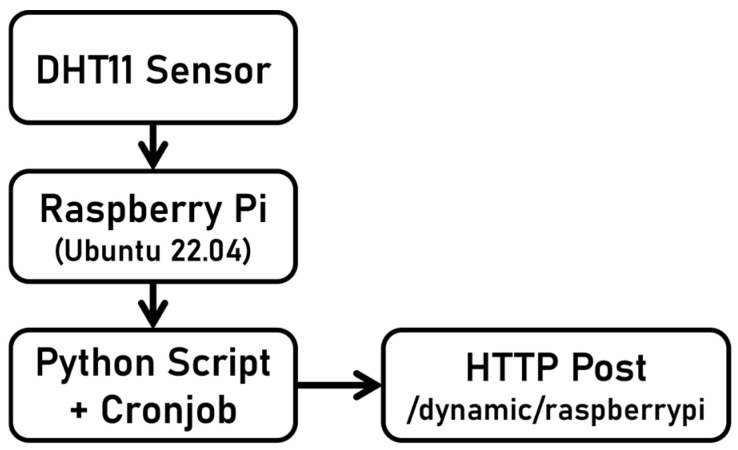
Raspberry hardware Pi Connection.

The Raspberry Pi was set up to run Ubuntu 22.04 and Python 3.6. To simplify the process of collecting system information and temperature and humidity data, a Python script was written. The script read the DHT11 sensor data connected on pin 7 (GPIO 4), with pin 1 (3.3 V) providing power and pin 6 providing ground (GND). The collected data were then sent to the defined API endpoint. The Python script was called using the cronjob scheduling system to facilitate the retrieval of data. Figure 3 and Figure 4 show the hardware connection for both devices.

#### 4.3.2. Trend Data

To analyze the results, JSON data were retrieved from the dynamic RESTful API for both the Raspberry Pi and ESP8266. Plots were then created using Matplotlib (v3.9.2) [47] to visualize the data. The dynamically generated tables for Raspberry Pi and ESP8266 in the database are displayed in Table 10 and Table 11, respectively. These tables contain a sample of the data stored in the database.

##### Raspberry Pi Data

**Figure 5:**
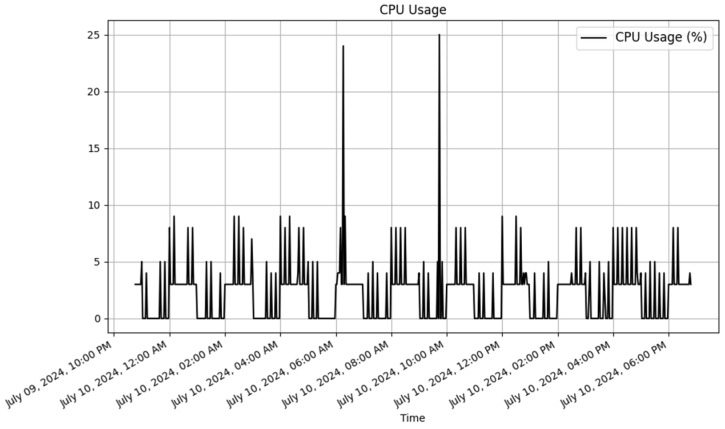
Raspberry Pi CPU usage graph.

**Figure 6:**
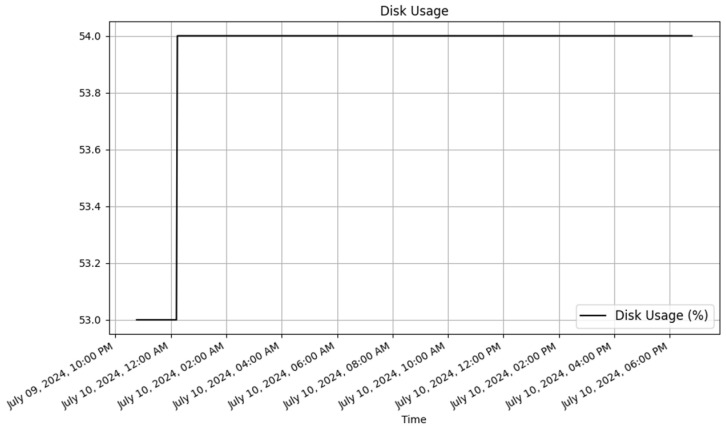
Raspberry Pi DISK usage graph.

**Figure 7:**
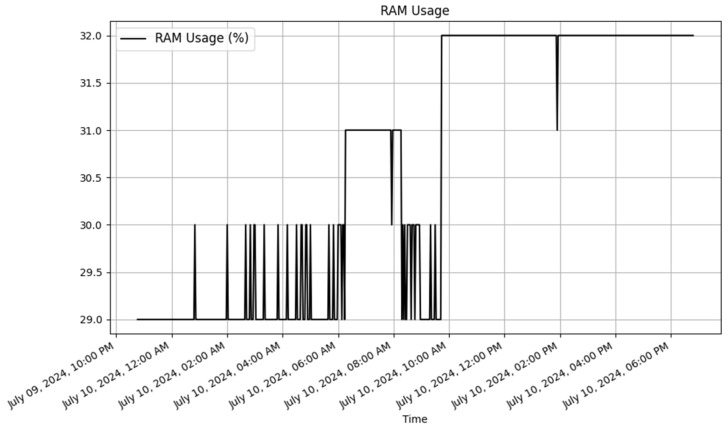
Raspberry Pi RAM usage graph.

The Raspberry Pi dynamically generated table with sample data is illustrated in Table 10. The data are presented in Table 10 and Figure 5, Figure 6 and Figure 7. These visual representations offered clear insights into the environmental conditions that were being monitored by the devices.

##### ESP8266 Data

**Figure 8:**
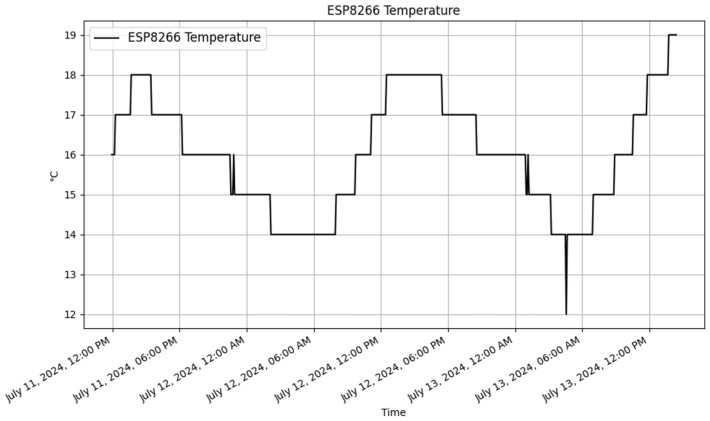
ESP8266 temperature graph.

**Figure 9:**
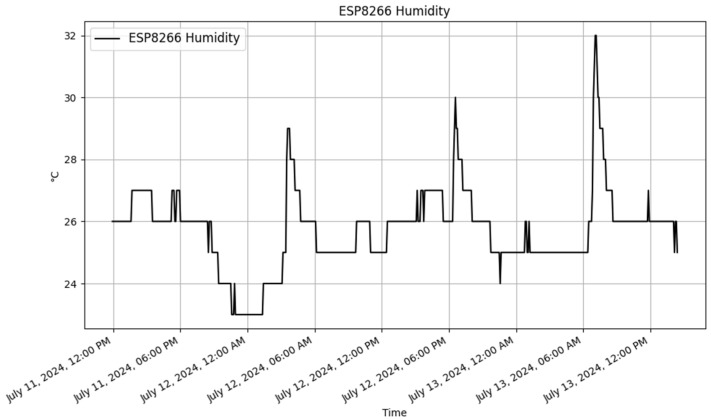
ESP8266 humidity graph.

The ESP8266 dynamically generated table with sample data is illustrated in Table 11. The data are presented in Figure 8 and Figure 9. These visual representations offered clear insights into the environmental conditions that were being monitored by the devices.

**Figure 10:**
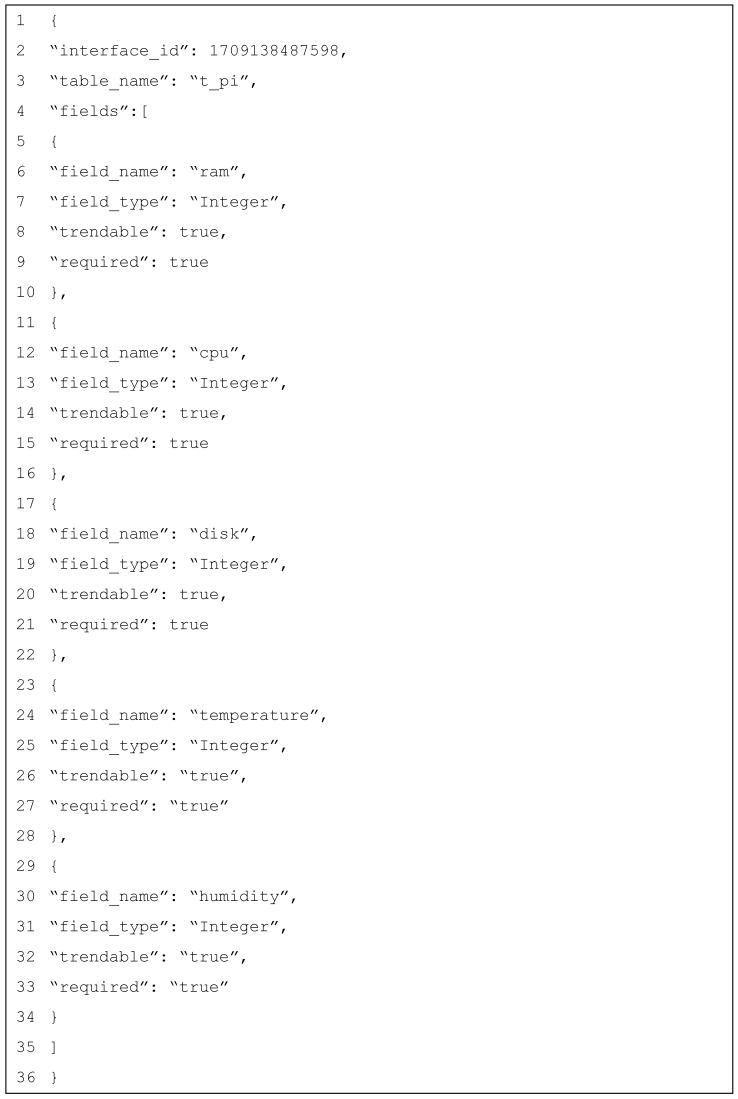
Raspberry Pi endpoint modification.

To assess the flexibility of the RESTful API, a temperature and humidity sensor was added on the Raspberry Pi. This was accomplished by updating the Raspberry Pi interface to include the temperature and humidity parameters. The JSON payload used to update the interface is illustrated in Figure 10.

**Figure 11:**
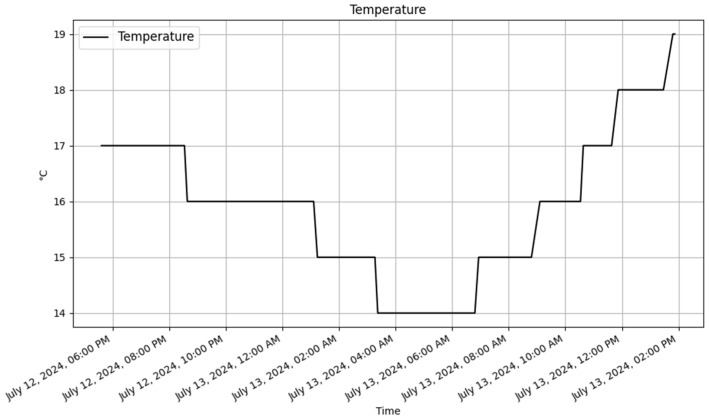
Raspberry Pi temperature trend.

**Figure 12:**
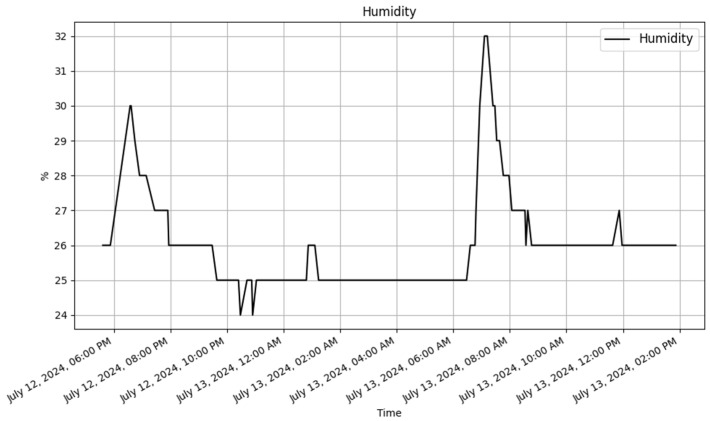
Raspberry Pi humidity trend.

The addition of the temperature and humidity sensor into the Raspberry Pi interface has improved its capabilities. The interface now contains real-time humidity and temperature data, providing useful insights and allowing for enhanced data analysis. This change highlights the API framework’s flexibility and adaptability, since it can easily extend its capabilities to include new devices, sensors, or additional parameters to existing ones. The ability to introduce such capabilities without disturbing the existing system architecture is a big advantage, demonstrating that the API is resilient and adaptable enough to manage a variety of changing requirements. The updated database table and sample records are illustrated in Table 12, while Figure 11 and Figure 12 display the new data for the Raspberry Pi, and they include the temperature and humidity values.

### 4.4. Performance Evaluation Results

#### 4.4.1. Functional Testing Results

The API endpoints were functionally tested to guarantee that they consistently returned correct responses and status codes. The major purpose was to validate the API’s functionality rather than evaluate its performance under high demand. The findings are summarized in Table 13.

All endpoints accomplished a 100% success rate, showing that the API processes requests consistently and delivers the desired results. This high-reliability level attests to the implemented functionality’s correctness and robustness. The /login endpoint was tested using POST requests to check user authentication. The successful handling of all samples confirmed that the authentication mechanism functions properly and regularly delivers valid responses.

The /dynamic endpoints were tested for various activities, including GET, PUT, POST, and Delete. Each action delivered correct data or successfully implemented the intended changes, confirming the functions’ correctness and stability. The /dynamic/interface/ endpoints were tested, and both POST and GET methods successfully added and retrieved data as expected.

The functional testing results confirm that all API endpoints are correctly implemented, consistently returning the appropriate responses and status codes. The system meets its functional requirements, ensuring a reliable experience

#### 4.4.2. Response Time Results

Response time testing of API endpoints was performed to assess the API’s performance in processing and responding to various requests. This testing calculated the average response time for each endpoint across several samples, offering insights into the API’s performance characteristics under different load scenarios. Table 14 provides an extensive summary of the results.

**Figure 13:**
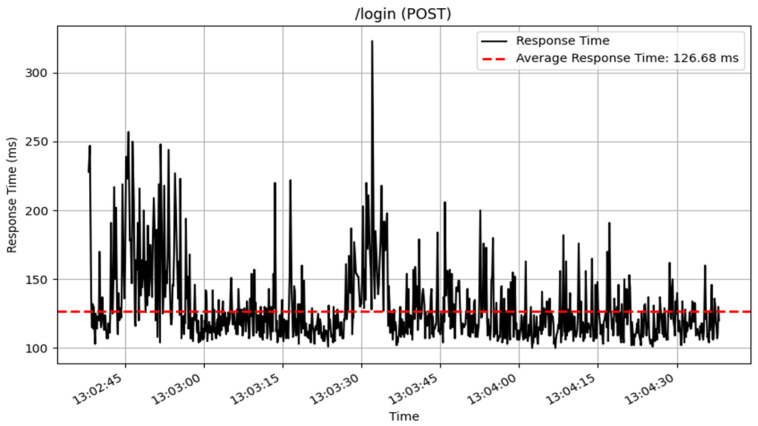
Login response time results.

The /login endpoint, which handles user authentication via POST requests, processed 908 samples, with an average response time of 127 milliseconds. The response times are represented in Figure 13. This result suggests that, while the login mechanism is reliable, there is room for further optimization to increase responsiveness.

**Figure 14:**
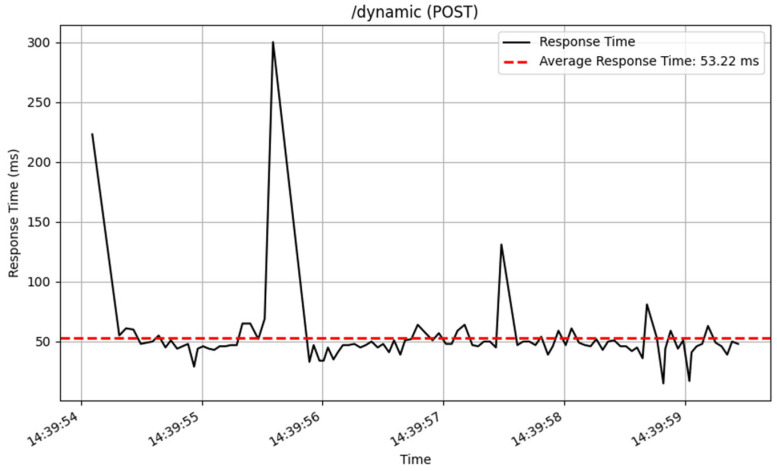
Add interface response time.

**Figure 15:**
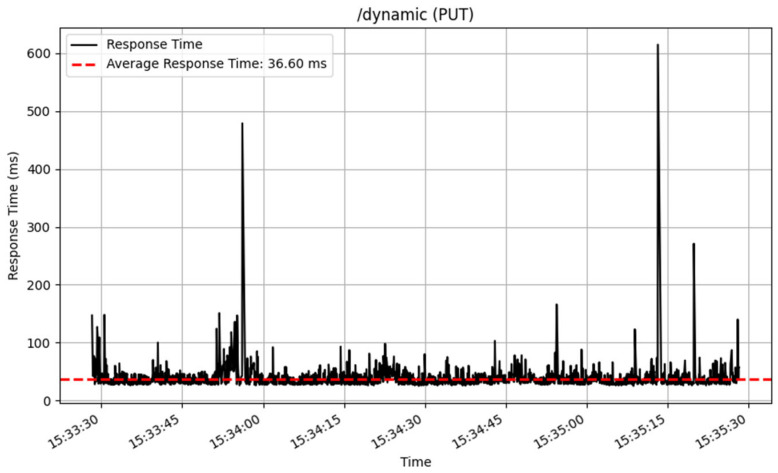
Update interface response time.

**Figure 16:**
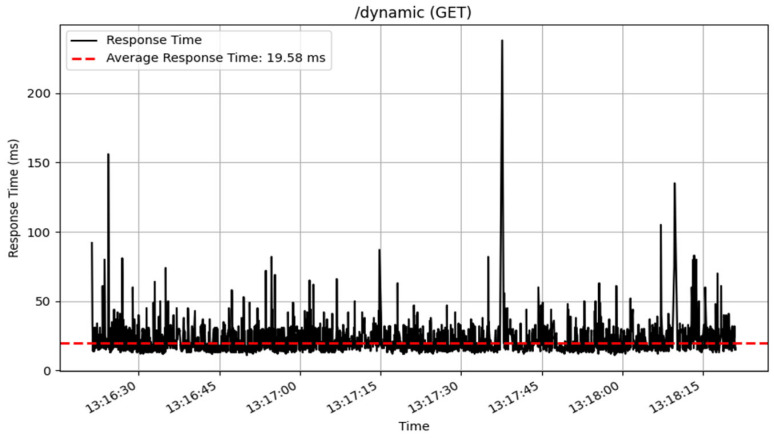
GET interface response time.

**Figure 17:**
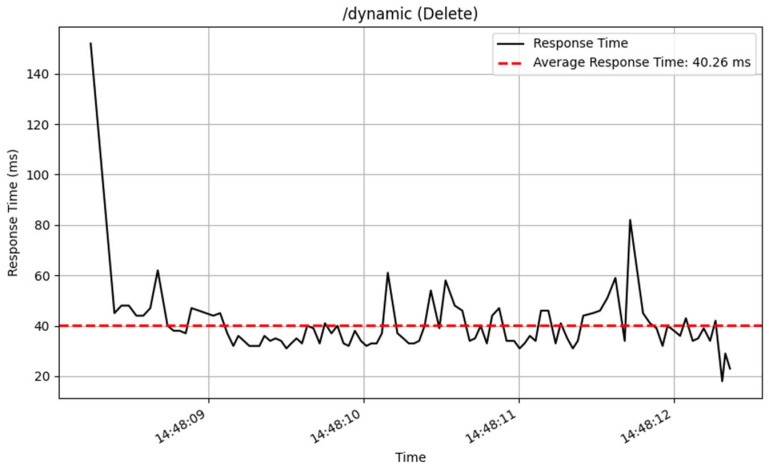
Delete interface response time.

The /dynamic endpoint performed differently depending on the method used. The GET method performed well, with an average response time of 19 milliseconds over 3872 samples, showing excellent speed in fetching dynamic content. The PUT method, which was used to update data, similarly performed well, with an average response time of 36 milliseconds for 2605 samples. The POST and DELETE methods performed consistently, processing a total of 100 samples with an average response time of 53 milliseconds. The response time of these CRUD method are illustrated in Figure 14, Figure 15, Figure 16 and Figure 17.

**Figure 18:**
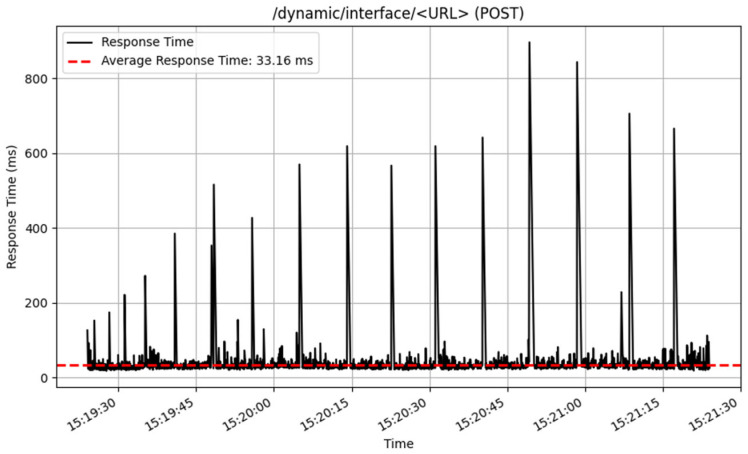
Add data to interface response time.

**Figure 19:**
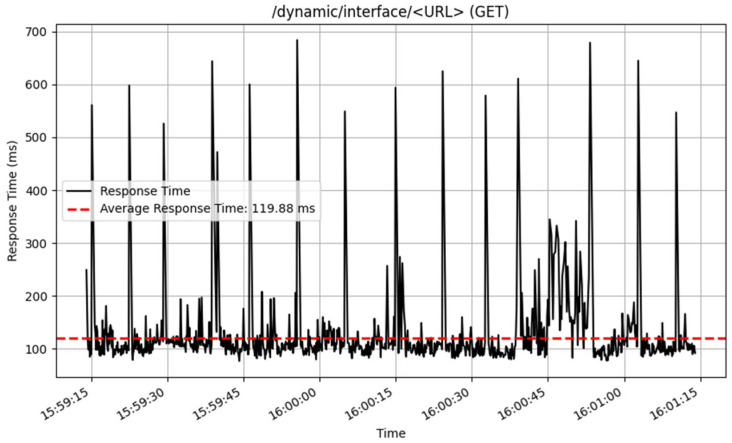
Get interface data response time.

The performance of the /dynamic/interface/ endpoints was also assessed, and the results are illustrated in Figure 18 and Figure 19. The POST method performed well, processing 3212 samples, with an average response time of 33 milliseconds. This proposes an effective handling of interface adding of endpoint history. In contrast, the GET technique for retrieving history data from dynamic interfaces returned an average response time of 119 milliseconds across 923 samples.

The response time testing confirms that the API endpoints are working as anticipated, with consistent and efficient performance over a wide range of requests. The results show that the system can handle various operations efficiently, contributing to a great user experience.

#### 4.4.3. Status Code Verification

During the status code verification process, the API endpoints were checked to ensure correct request and response handling. The /login (POST) endpoint gave response codes 200 for successful logins, 401 for unauthorized access, 404 for not found, and 422 for validation issues. The /dynamic endpoint (POST) gave response codes 200 for successful creation, 400 for bad requests, and 422 for validation errors, yet the /dynamic (GET) endpoint constantly returned 200 for successful retrieval.

The /dynamic (PUT) and /dynamic (DELETE) endpoints both returned 200 for successful updates and deletions, 400 for invalid requests, and 422 for validation errors. The /dynamic/interface/<URL> (POST) endpoint returned 200 for successful data insertion and 400 for invalid requests, whereas /dynamic/interface/<URL> (GET) returned 200 for successful data retrieval. These findings demonstrate that the API handled queries correctly across a variety of contexts. Table 15 summarizes the verified status codes for all the endpoints.

#### 4.4.4. Load Testing Results

The load test was executed over a duration of 141 s, during which 4732 requests were generated using a single thread with consistent API key authentication. To obtain accurate application performance results, the Nginx rate limit of 10 requests per second was bypassed, as evidenced by the initial throughput exceeding this limit, allowing the test to stress the API beyond the configured threshold and evaluate its underlying behavior under high load.

Analysis of the raw data revealed key metrics across the 4732 samples. A total of 4585 requests succeeded (96.89%), while 147 failed, resulting in a 3.11% error rate, all attributed to 404 “Not Found” errors. Response times ranged from a minimum of 5 ms to a maximum of 760 ms, averaging 88.69 ms, indicating generally efficient performance with some variability. The overall throughput was 9.83 requests/s, slightly below the target rate of 10 requests/s, but the per-minute analysis revealed periods of significantly higher throughput due to the rate limit bypass.

To examine performance trends, the data were aggregated into 1 min intervals, yielding three bins over the 141 s duration. The first minute recorded 4026 samples, with an average response time of 90 ms (estimated based on the overall average and distribution), 125 errors (3.1% of this bin), and a throughput of 67.1 requests/sec, far exceeding the configured rate limit of 10 requests/s due to the intentional bypass. The second minute included 690 samples, with an average response time of 85 ms, 21 errors (3.04%), and a throughput of 11.5 requests/s, closer to the target rate. The final 20 s, forming the third bin, had 16 samples, an average response time of 82 ms, 1 error (6.25%), and a throughput of 0.8 requests/s. The high initial throughput confirms that bypassing the rate limit allowed JMeter to generate a burst of requests, which later stabilized as the test progressed, though it did not sustain the load consistently.

Response times averaged 88.69 ms, which is highly efficient for an API, but the maximum of 760 ms highlights significant outliers, likely corresponding to the 404 errors or server-side delays under the excessive load enabled by the rate limit bypass. The minimum response time of 5 ms aligns with the rapid responses observed for failed requests, suggesting the server quickly identified unavailable resources. The error rate of 3.11%, driven by 147 instances of 404 errors, showed clustering, with 125 errors in the first minute, possibly due to server-side issues exacerbated by the initial high request volume of 67.1 requests/sec.

The overall average throughput of 9.83 requests/s demonstrates that the server maintained a rate close to the target on average, but the per-minute analysis reveals an initial peak of 67.1 requests/s, dropping to 11.5 requests/s and then 0.8 requests/s. This pattern indicates that bypassing the rate limit allowed an early surge in requests, stressing the application beyond its normal operational limits. The decline in throughput toward the end of the test suggests that the load was not sustained consistently, potentially due to the test’s short duration or a lack of throttling in the JMeter setup.

**Figure 20:**
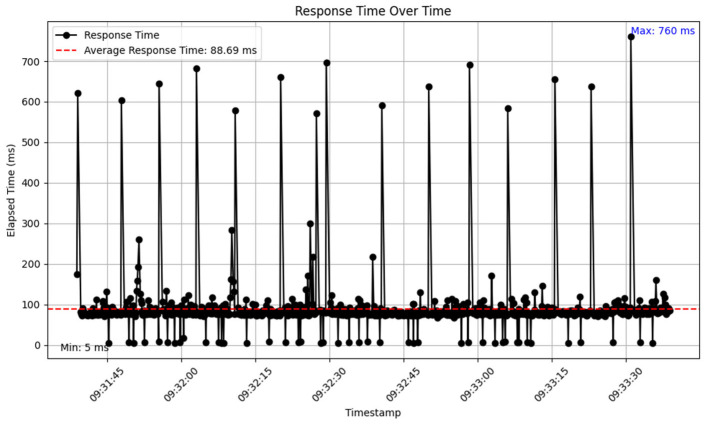
Load test trend data.

Bypassing the rate limit, as confirmed by the first minute’s throughput of 67.1 requests/s, allowed the test to reveal the API’s performance under stress, highlighting the server’s capacity to handle bursts of traffic. However, this also exposed vulnerabilities, as the 3.11% error rate, driven by 404 errors, coincided with response time spikes, such as the maximum of 760 ms, indicating server-side issues like resource unavailability under high load. The efficient average response time of 88.69 ms demonstrates the API’s capability to handle requests quickly, but the variability in response times points to potential bottlenecks that need addressing. The test’s inability to sustain a steady load throughout highlights a design limitation in the JMeter configuration, likely due to inadequate throttling or loop control, leading to an initial burst and subsequent decline in request rate over the 141 s duration. The performance metrics captured during the test are illustrated in Table 16 and Figure 20:

## 5. Conclusions

This study aimed to develop and evaluate a dynamic RESTful API framework tailored for flexible, scalable, and maintainable IoT deployments, addressing the critical need for adaptive architectures in the IoT environment. Using Python and FastAPI, the framework enabled dynamic endpoint generation and runtime schema adaptability, allowing smooth system expansion without modifying the core application structure, a key objective of this work. Load testing on a VPS with 2 vCPUs and 2 GB RAM provided insights into performance under varying conditions. The framework achieved an average throughput of 9.83 requests/s across 4732 requests over 141 s, peaking at 67.1 requests/s, with an average response time of 88.69 ms. However, this led to a 3.11% error rate and response time spikes up to 760 ms, highlighting server-side bottlenecks due to resource constraints, as analyzed in Section 4.4.4.

The contribution of this work lies in advancing IoT system design by demonstrating how dynamic RESTful APIs can address the limitations of static architectures, a challenge well documented in prior literature. Unlike traditional solutions such as Python frameworks like Flask and Django, which support dynamic routing (e.g., /data/<device_id>) but rely on static, hardcoded schemas, GraphQL, which depends on static schemas and heavy resolver logic, gRPC, which requires precompiled protocols limiting web interoperability, or API Gateways, which lack runtime schema changes and introduce latency, this framework dynamically generates both endpoints and data structures at runtime. This capability is particularly impactful in IoT scenarios requiring the addition of new parameters or I/O components such as environmental monitoring (e.g., integrating new temperature sensors) or industrial automation (e.g., adding machine sensors) where immediate monitoring is enabled without system downtime or redeployment. The framework’s Dockerized deployment further enhances its scalability and portability, distinguishing it from static API frameworks that struggle with the heterogeneity and scale of IoT deployments.

In the future, the framework could be enhanced by incorporating a pub/sub protocol like MQTT to support broader communication models, particularly for event-driven architectures, which would improve its suitability for diverse IoT use cases. Additionally, introducing a dedicated alert and notification system as a separate container could ensure efficient event processing without interfering with the existing implementation, further strengthening the framework’s capabilities. Moreover, optimizing server-side resource allocation to reduce the error rate and response time variability, as well as extending the framework to support multi-region deployments for enhanced global scalability, are planned improvements. Ultimately, this work advances the development of RESTful APIs by offering a robust solution for dynamic IoT infrastructures across various industries, with clear pathways for further enhancement.

## Figures and Tables

**Table 1:** VPS specifications.

Component	Specification
CPU	2 virtual CPUs (vCPUs)
Memory	2 GB RAM
Storage	40 GB SSD
Operating System	Ubuntu 22.04 LTS (64-bit)

**Table 2:** Summary of the dynamic interfaces.

Endpoint	Method	Description
/dynamic	POST	Create a new dynamic interface
/dynamic	GET	Retrieve dynamic interfaces
/dynamic	PUT	Update a dynamic interface
/dynamic	DELETE	Delete a dynamic interface
/dynamic/<URL>	POST	Add data to a dynamic endpoint
/dynamic/interface/<URL>	GET	Retrieve data from a dynamic endpoint

**Table 3:** Add Interface API Details.

Category	Field	Value	Type
Request	URL	/dynamic	
	Method	POST	
	Content-Type	application/json	
	Payload		JSON
	interface_url		string
	interface_description		string
	table_name		string
	fields		array
	Inside ‘fields’ array		
	field_name		string
	field_type		string
	trendable		boolean
	required		boolean
Response	URL	/dynamic	
	Status Code	200	
	Content-Type	application/json	
	Payload		JSON
	interface_id		integer
	interface_url		string

**Table 4:** Get interface request and response.

Category	Field	Value	Type
Request	URL	/dynamic	
	Method	GET	
	Content-Type	application/json	
	Payload		JSON
	interface_id		integer
Response	URL	/dynamic	
	Status Code	200	
	Content-Type	application/json	
	Payload		JSON
	interface_id		integer
	interface_url		string
	interface_description		string
	table_name		string
	fields		array
	Inside ‘fields’ array		
	field_name		string
	field_type		string
	trendable		boolean
	required		boolean

**Table 5:** Update interface request and response.

Category	Field	Value	Type
Request	URL	/dynamic	
	Method	PUT	
	Content-Type	application/json	
	Payload		JSON
	interface_id		integer
	interface_description		string
	fields		array
	Inside ‘fields’ array		
	field_name		string
	field_type		string
	trendable		boolean
	required		boolean
Response	URL	/dynamic	
	Status Code	200	
	Content-Type	application/json	
	Payload		JSON
	success		string

**Table 6:** Delete interface request and response.

Category	Field	Value	Type
Request	URL	/dynamic	
	Method	DELETE	
	Content-Type	application/json	
	Payload		JSON
	interface_id		integer
Response	URL	/dynamic	
	Status Code	200	
	Content-Type	application/json	
	Payload		JSON
	success		string

**Table 7:** Add interface record request.

Category	Field	Value	Type
Request	URL	/dynamic/<URL>	
	Method	POST	
	Content-Type	application/json	
	Payload		JSON
	<dynamic_field_1>		integer
	<dynamic_field_2>		string
	<dynamic_field_3>		boolean
	…	…	…
Response	URL	/dynamic	
	Status Code	200	
	Content-Type	application/json	
	Payload		JSON
	success		string

**Table 8:** GET interface history request and response.

Category	Field	Value	Type
Request	URL	/dynamic/interface/<URL>	
	Method	GET	
	Content-Type	application/json	
	Payload	None	
Response	URL	/dynamic/interface/<URL>	
	Status Code	200	
	Content-Type	application/json	
	Payload		JSON
	<dynamic_field_1>		integer
	<dynamic_field_2>		string
	<dynamic_field_3>		boolean
	…	…	…

**Table 9:** Dynamic interface table information.

ID	Interface_ID	Interface_URL	Interface_Description	Table_Name	Fields	Interface_Owner
4	1709138487598	/raspberrypi	Endpoint for the Pi	t_pi	{“{“field_name”: “ram”, “field_type”: “Integer”, “trendable”: true, “required”: true}”,”{“field_name”: “cpu”, “field_type”: “Integer”, “trendable”: true, “required”: true}”,”{“field_name”: “disk”, “field_type”: “Integer”, “trendable”: true, “required”: true}”,”{“field_name”: “epoch_time”, “field_type”: “Integer”, “trendable”: false, “required”: false}”}	2
39	1709830425232	/esp	Endpoint for the esp8266 weather station	t_esp	{“{“field_name”: “temperature”, “field_type”: “Integer”, “trendable”: true, “required”: true}”,”{“field_name”: “humidity”, “field_type”: “Integer”, “trendable”: true, “required”: false}”,”{“field_name”: “epoch_time”, “field_type”: “Integer”, “trendable”: false, “required”: false}”}	2

**Table 10:** Raspberry Pi dynamic database table.

ID	Ram	CPU	Disk	Epoch_Time
38710	31	0	51	1,716,038,767
38708	31	0	51	1,716,038,526
38706	31	0	51	1,716,038,286
38703	31	0	51	1,716,037,926

**Table 11:** Esp8266 dynamic database table.

ID	Temperature	Humidity	Epoch_Time
13332	24	32	1,716,039,303
13331	24	32	1,716,039,007
13330	24	33	1,716,038,713
13329	24	32	1,716,038,403

**Table 12:** Raspberry Pi updated database table.

ID	Ram	CPU	Disk	Epoch_Time	Temperature	Humidity
38710	31	0	51	1,716,038,767	24	33
38708	31	0	51	1,716,038,526	24	33
38706	31	0	51	1,716,038,286	24	33
38703	31	0	51	1,716,037,926	24	33

**Table 13:** API functional test results.

Endpoint	Method	# Samples	Success Rate (%)
/login	POST	908	100
/dynamic	POST	100	100
/dynamic	GET	3872	100
/dynamic	PUT	2605	100
/dynamic	DELETE	100	100
/dynamic/interface/<URL>	POST	3212	100
/dynamic/interface/<URL>	GET	923	100

**Table 14:** API response time test results.

Endpoint	Method	# Samples	Average Response Time (ms)
/login	POST	908	127
/dynamic	POST	100	53
/dynamic	GET	3872	19
/dynamic	PUT	2605	36
/dynamic	DELETE	100	53
/dynamic/interface/<URL>	POST	3212	33
/dynamic/interface/<URL>	GET	923	119

**Table 15:** Status code verification results.

Endpoint	Method	Status Codes Verified
/login	POST	200, 401, 404, 422
/dynamic	POST	200, 400, 422
/dynamic	GET	200
/dynamic	PUT	200, 400, 422
/dynamic	DELETE	200, 400, 422
/dynamic/interface/<URL>	POST	200, 400
/dynamic/interface/<URL>	GET	200

**Table 16:** Load testing results.

Metric	Value
Total Requests	4732
Successful Requests	4585
Failed Requests	147 (3.11% error rate)
Average Response Time	88.69 ms
Max Response Time	760 ms
Min Response Time	5 ms
Throughput	9.83 requests/s

## Data Availability

The raw data supporting the conclusions of this article will be made available by the authors on request.

## Abbreviations

The following abbreviations are used in this manuscript:

API	Application Programming Interface
IoT	Internet of Things
CRUD	Create, Read, Update, Delete
GPIO	General Purpose Input/Output
